# Influence of TiO_2_ Nanoparticles on the Resistance of Cementitious Composite Materials to the Action of Fungal Species

**DOI:** 10.3390/ma14164442

**Published:** 2021-08-08

**Authors:** Andreea Hegyi, Elvira Grebenişan, Adrian-Victor Lăzărescu, Vlad Stoian, Henriette Szilagyi

**Affiliations:** 1 NIRD URBAN-INCERC Cluj-Napoca Branch, 117 Calea Floreşti, 400524 Cluj-Napoca, Romania; elvira.grebenisan@incerc-cluj.ro; 2 IOSUD-UTCN Doctoral School, Technical University of Cluj-Napoca, 15 Daicoviciu Street, 400020 Cluj-Napoca, Romania; 3 Department of Microbiology, Faculty of Agriculture, University of Agricultural Sciences and Veterinary Medicine Cluj-Napoca, 3-5 Calea Mănăştur, 400372 Cluj-Napoca, Romania

**Keywords:** self-cleaning cementitious composites, TiO_2_ nanoparticles, photocatalysis, antifungal effect

## Abstract

The development of mold films on the cement surfaces of buildings is a health and safety problem for the population, aesthetic but also in terms of their durability. The use of specific performance of cementitious composites containing TiO_2_ nanoparticles, photoactivated by UV radiation, can be a viable solution to mitigate to eliminate these problems. The experimental studies presented aim to analyze the capacity to inhibit the development of mold type *Aspergillus* and *Penicillium* on the surface of composite materials with nano-TiO_2_ content and the identification of the optimal range of nanomaterial addition. The identification and analysis of the inhibition halo (zone with a biological load of maximum 1–10 colonies of microorganisms) confirmed the biocidal capacity of the cementitious composites, but also indicated the possibility that an excess of TiO_2_ nanoparticles in the mixture could induce a development of cell resistance, which would be unfavorable both in terms of behavior and in terms of cost.

## 1. Introduction

Worldwide, there is a change in the lifestyle of the population, with many of the daily activities moving from outside to inside [1]. At the same time, the need for the development of so-called “smart” building materials has been identified, with superior performances in terms of durability and meeting sustainability criteria.

It is currently known that the development of microorganisms (molds, bacteria, viruses, algae, lichens, mites) on the surfaces of buildings has negative effects on the health of the population, especially if the development takes place on the surfaces of indoor living spaces, on the sustainability and safety in operation of buildings and on the degree of environmental pollution. The overall negative impact on the health of users is recognized worldwide as ‘sick building syndrome (SBS)’, manifesting itself in the population operating, partially or totally, inside buildings affected by micro-organisms deposits, as a result of degradation of indoor air quality by contamination with spores and toxins [1,2,3,4,5,6,7]. The most common mycotoxins whose presence has been identified in indoor air but also in the body of the population that stayed in the contaminated environment are *Ochratoxin* (OCT), *aflatoxin B1*, *Trichothecene* [8,9,10,11,12,13,14]. These microtoxins are also produced by molds of the *Aspergillus* and *Penicillium* genera and are genotoxic, immunotoxic, hepatotoxic, mutagenic and potentially carcinogenic [8,15]. Epidemiological studies have shown that in more than 15% of cases of asthma diagnosed in children, one of the main causes was exposure to mold spores in the air inside the rooms [16]. On the other hand, at the same time, there are negative effects on the health of the built environment, since, in addition to an unpleasant appearance that induces cleaning costs and preservation of aesthetic value, there are also costs for maintenance and repairs necessary with greater frequency, as a result of favoring the development of biological corrosion, initially at the surface and subsequently penetrating deeper into the constructed element [17,18,19]. All these costs are both financial (labor, materials, interruption of construction during repairs, etc.) and environmental impact costs (air pollution–greenhouse gases, dust, volatile organic products–VOCs; water and soil pollution–wastewater, solid waste, noise pollution and consumption of raw materials and energy needed to manufacture other building materials to replace those affected).

After plastics, concrete is the building material most commonly affected by mold [20]. According to the literature, among the most common mold species affecting constructions belong to the families *Cladosporium*, *Acremonium*, *Alternaria*, *Periconia*, *Curvularia*, *Stemophylium*, *Penicillium*, *Aspergillus* [1,7,20]. A study conducted in the USA indicated that species from the families *Penicillium* and *Aspergillus* were found in more than 50% of the cases of commercial buildings and residential buildings analyzed, especially in conditions of high humidity and low direct solar radiation [21,22]. 

Therefore, it is desirable to identify solutions for increasing the degree of indoor air hygiene, durability of constructions and printing a self-maintenance capability. Harnessing the photocatalytic process of TiO_2_ nanoparticles proposes a simple and promising technology for inhibiting the development of mold film on the surface of cementitious materials. Thus, by simply exploiting sunlight, it becomes an alternative to the use of chemical disinfectants [23,24,25,26,27], especially since buildings have large areas, exposed to solar radiation for much of the day.

These cementitious composites, intended for the construction sector, become “intelligent” by embedding TiO_2_ nanoparticles in their matrix, acquire and show self-cleaning capacity under the action of environmental factors (natural, solar, or artificial UV irradiation and rain). Currently, there is research reported in the literature indicating that, even under low–intensity light irradiation conditions (the situation of the interior surfaces of buildings), a natural light intensity of ∼10 MW cm^−2^ or a simple neon bulb is sufficient to provide a sufficient amount of UV activation energy to allow photoactivation and therefore the self-cleaning effect of the composite cementitious surface [28].

The self-cleaning capacity of cementitious composites with nano-TiO_2_ content is the sum of two phenomena, both due to the behavior of TiO_2_ nanoparticles under UV radiation: on the one hand, a superhydrophilicity of the cementitious surface develops which, when in contact with water, allow for the formation of a continuous film that will wash away the more effective the particles deposited on the surface, and, on the other hand, are catalyzed oxidation reactions in the molecules of the organic in nature, they are transformed into the products, the organic molecule is smaller and more easily washed off, and more easily destroyed in turn by means of a new process for the oxidation to CO_2_ and H_2_O [17,29,30,31,32,33,34,35,36,37]. The literature [17] indicates that TiO_2_ nanoparticles, alone, lose their hydrophilicity influence property as soon as the action of UV radiation ceases, but in combination with SiO_2_ in cement, the UV-activated effect extends even more days of darkness (total lack of UV radiation).

The mechanism underlying the increase in hydrophilicity of cementitious composite surfaces with TiO_2_ nanoparticles under conditions of exposure to the action of UV rays can be explained on the basis of the increase in hydroxyl groups (OH^−^), phenomenon identified by X–ray photoemission spectroscopy (XPS), Fourier Transform Infrared Spectroscopy (FTIR) or nuclear magnetic resonance (MRI) [17,38,39,40]. The transition of the surface, under the influence of UV radiation, into a metastable thermodynamic state is the result of the coexistence of a memory of molecular water and dissociated water. In general, under the action of UV rays, titanium dioxide being a semiconductor with a band gap of about 3.0 eV, by absorbing energy, generates electrons (e^−^) and holes (h^+^). Electrons tend to reduce the Ti (IV) cation to Ti (III) ions, and holes oxidize O_2_^−^ anions. This process will release oxygen creating gaps on the surface of titanium dioxide, gaps that give the possibility of binding water molecules with the release of hydroxyl (OH^−^) groups. In the case of cementitious composite surfaces containing TiO_2_, the literature also indicates [17,41] that photogenerated holes (h^+^) increase the length of the bonds within the TiO_2_ network bringing the surface to a metastable state that allows the adsorption of molecular water, simultaneously with the formation of new hydroxyl groups and the release of a proton. Research believes that these generated hydroxyl groups are less thermodynamically stable, therefore the surface will allow flattening of the water drop to cover a larger area for stabilization purposes [17,41,42]. 

As for the mechanism of oxidation of organic molecules, with energy supplied by UV radiation, greater than the valence band difference of TiO_2_, electron pairs (e^−^) and voids (h^+^) are generated, which, react with O_2_ and H_2_O forming anionic radicals (O_2_^−^) and (OH^−^). These oxidative species (h^+^,∙(O_2_^−^) and (OH^−^)) are all highly reactive and contribute to the destruction of the cells of microorganisms [43,44]. Since the gap oxidation capacity (h^+^) is greater than the electron reduction capacity (e^−^), and on the surface of the photocatalyst there is a single layer of adsorbed H_2_O molecules, hydroxyl groups (OH^−^) are formed. These highly oxidizing hydroxyl groups react with organic molecules producing peroxyl free radicals, which will react with molecular oxygen in a chain of reactions up to the final products CO_2_ and H_2_O and contribute to cell membrane rupture [45,46,47]. On the other hand, the electrons (e^−^) reduce oxygen to the free radical O_2_^−^ which will react with the resulting peroxide molecules during the reaction between the hydroxyl groups and the organic molecules, ultimately also leading to a chain of reactions up to the final products CO_2_ and H_2_O [28,48,49,50,51,52,53,54]. Research indicates a wavelength of TiO_2_ photoactivation incident radiation that produces strong destruction at the cellular level in the range of 320–400 nm [55,56,57,58,59]. In this context, there is the destruction of the integrity of the cell membrane and the induction of disorder at the level of the cell, which is the explanation of the biocidal effect [45,46,60,61,62,63].

Although the potential benefits of TiO_2_ nanoparticles have been known since 1921 [64], the ability to destroy microorganisms has only been documented after 65 years [65,66,67]. Currently, it is known that the analgesic and antifungal activity of nano–TiO_2_ is evident on 11 genera of filamentous fungi, 3 yeasts, 2 amoeba, 1 Apicomplexan, 1 diplomonad, 1 ciliate and 7 algae, including 1 diatom, fungal spores being generally more resistant than vegetative forms [45,68,69,70,71,72,73,74,75,76,77,78].

As a result of the above presented, it is appreciated that it is of major interest to develop solutions to increase and improve the resistance of the surface of the constructions to the development of mold, thus contributing to the increase in the durability and sustainability of the constructions, whit the aim to achieve the goals of the concept of Sustainable Development.

Therefore, the aim of this study is to assess the capacity of the increase in the resistance to the action of mold of white Portland cement cementitious composites, with various amounts of TiO_2_ nanoparticles addition (up to 6%), and to identify the range of the concentration of nanoparticles in relation to the amount of cement, which provides effectiveness, from the point of view of the resistance of biological tissue.

## 2. Materials and Methods

### 2.1. Cementitious Composites Based on White Portland Cement with Nano-TiO_2_ Addition

Cementitious composites paste, with TiO_2_ nanoparticles addition, was prepared and conditioned according to Table 1. P1 mixture is considered the control sample (0% nano-TiO_2_ content). The TiO_2_ nanoparticles used were commercial AEROXIDE^®^ TiO_2_ P25 by Evonik Degussa Industries AG, Hanau, Germany. According to the manufacturer’s Data Sheet, these TiO_2_ nanoparticles are characterized by an average particle size of 21 nm, with a specific surface of 35–65 m^2^/g, 99.5% purity, containing more than 70% anatase, with a minor amount of rutile and a small amount of amorphous phase anatase and rutile crystallites, both phases playing an important role in industrial applications and contribute to the photoactivation mechanism [4].

Research carried out previously and in accordance with some specifications in the literature [8,9,20,61,63], showed that TiO_2_ nanoparticles have a very small density, which causes a large volume of material added to the initial volume of cement. It is therefore not possible to obtain a cementitious paste enriched with TiO_2_ nanoparticles under conditions of constant preservation of the amount of preparation water, without affecting the consistency and workability of the fresh material and, implicitly, the properties of the hardened material. Accordingly, the standard consistency of the paste, determined with the Tetmayer probe, was established as a constant parameter, and depending on it, the mixing water was determined for each individual cement/TiO_2_ nanoparticle ratio.

The cementitious composite paste was poured into patterns, obtaining rectangular plates from which samples were extracted in the form of discs with a diameter of 17.4 mm, respectively, plates with dimensions 24 mm × 30 mm.

These samples were subjected to photoactivation by holding for 24 h under the action of UV rays, using a light source with emission in the 400–315 nm spectrum, corresponding to the UVA band, located at a distance of 10 cm above the surface of the samples, which determined a luminous flux intensity of 860 lux.

After photoactivation, the circular samples were exposed to an environment contaminated with *Aspergillus niger* and the rectangular samples to an environment contaminated with *Penicillium notatum* spores.

### 2.2. Exposure of Cementitious Composite Samples Based on White Portland Cement with Nano-TiO_2_ Addition to an Environment Contaminated with Mold Spores

In the laboratory, Petri dishes, φ 9 cm, were prepared, in which a nutrient subtract of Potato Dextrose Agar (PDA) was placed, known as a medium conducive to the growth of mold crops. The nutrient PDA substrate was prepared by dissolving the dehydrated substance in hot water (39 g/l) and pouring the resulting solution into the Petri dish. After cooling and curing of the PDA solution, the Petri dishes were sterilized 24 h under the action of UV rays.

For testing the resistance of the cementitious composites to colonization with molds, two species of mold were used, extracted from selected homes. Both species were isolated, purified and maintained in the collection of microorganisms of the Microbiology discipline of the Faculty of Agriculture within USAMV Cluj–Napoca, Romania. Pure cultures of *Aspergillus niger* (FAg18003) and *Penicillium notatum* (Fag19002) have been used as a source of inoculum in Petri dish contamination experiments. Solutions of spores of *Penicillium notatum*, respectively, of *Aspergillus niger* were prepared form mold cultures by introducing 2 loops of 10 µL of biological material in 1 mL of distilled water. These types of mold were chosen for the study because of the high frequency with which they are encountered on the surface of the built environment, both inside and outside buildings, and because of the toxicity of emanated aflatoxins and the high frequency with regard to the reporting of population diseases arising from exposure to them [7,16,20,21].

In each Petri dish with sterilized PDA nutrient substrate, 1.5 mL of mold spore suspension was applied and distributed so that the entire surface was covered with PDA, after which the photoactivated cementitious composite sample with nanoparticle addition was centrally placed under handling conditions that did not allow cross-contamination of the system. Subsequently, on the cementitious composite sample, 0.5 mL of spore suspension was applied, and the Petri dish lid was placed, the entire system was isolated on the edge with sealing tape to prevent cross-contamination.

For each mold species, a growth system was also prepared that did not contain a cementitious composite sample. By continuously and evenly distributed growth of the mold film that has covered the entire surface of the nutrient medium, they constitute the viability control systems of the spore solutions used.

**Figure 1:**
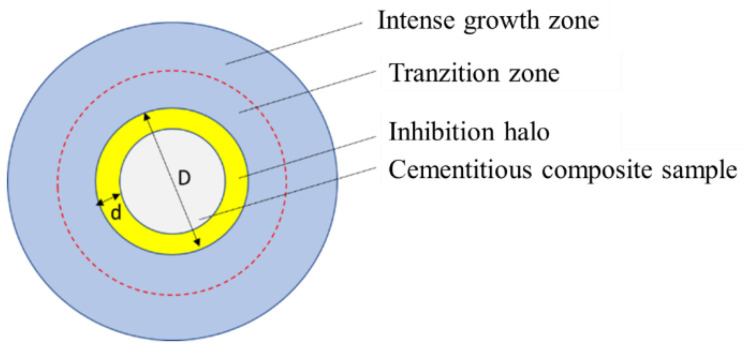
Evaluation of indicators principle.

The growth systems thus prepared were placed in laboratory conditions, (23 ± 2 °C), (65 ± 5%) RH, natural lighting conditions. At regular intervals (24 h) the samples were examined visually and microscopically to identify signs of growth/development of the biological material and the development of the inhibition halo (the zone in the immediate vicinity of the cementitious sample, with a biological load of maximum 1–10 colonies of microorganisms) was followed, recording its diameter (D), as an average of at least 5 measurements (Figure 1), using a Leica DMC2900 Stereomicroscope (Leica Microsystems^®^, Wetzlar, Germany).

Due to the difference in shape between the two sets of samples, the distance from the edge of the composite sample to the outer limit of the inhibition halo (d) was also measured simultaneously with the measurement of the diameter of the inhibition halo. This distance (d), expressed as average of minimum of five measurements, was considered a useful indicator in the resistance comparison test, expressed by the radius of action of the mold growth inhibition effect, which each cementitious composition with the addition of nano-TiO_2_ shows in an environment contaminated with *P. notatum* spores, vs. environment contaminated with *A. niger* spores, eliminating the variable introduced by the size of the composite sample.

### 2.3. Analysis of the Experimental Results

For each exposure time and for each mixture of cementitious composite containing nano–TiO_2_ the following indicators were calculated:

- The percentage value, relative to the control sample, which represents the larger the diameter of the inhibition halo compared to the control’s inhibition diameter, (CPCM) calculated according to Equation (1):
  (1) $$\mathrm{CPCM}=\frac{D_{days}^{\%TiO2}-D_{days}^{control}}{D_{days}^{control}}*100\;\left(\%\right)$$
- Reduction in the diameter of the inhibition halo over time (RDT) calculated according to Equation (2):
  (2) $$\mathrm{RDT}=\frac{D_{2}^{\%TiO2}-D_{i}^{\%TiO2}}{D_{2}^{\%TiO2}}*100\;\left(\%\right)$$
  where: $D_{days}^{\%TiO2}$—the diameter of the sample inhibition halo with % TiO_2_ at 2, respective 3 days exposure in contaminated environment; $D_{days}^{control}$—the diameter of the inhibition halo of the control sample, with 0% TiO_2_, at 2, respectively 3 days exposure in contaminated environment; $D_{2}^{\%TiO2}$—sample inhibition halo diameter with % TiO_2_ at 2 days exposure in contaminated environment; $D_{i}^{\%TiO2}$—sample inhibition halo diameter with % TiO_2_ at i > 2 days exposure in contaminated environment.

### 2.4. Statistical Analysis

The data analysis was performed with different packages available in the RStudio software, on the R platform. The basic statistics were performed with the “psych” package, the averages and the standard error observed in each experimental variant analyzed were extracted. To assess the presence of significant differences between variants, the ANOVA test from the “agricolae” package was applied. Positive ANOVA tests allowed post-hoc exploration with the LSD test available in the same package. LSD analysis was applied dynamically; completely contaminated mold variants were removed from the analysis. This approach wanted to highlight the real differences between the tested mixtures that were successful in blocking the development of fungi. We preferred the LSD test for its potential to compare multiple variants, based on their differences at *p* < 0.05. This permits the usage of both means ± s.e., followed by letters which indicate the significant differences based on international standards for post-hoc tests. The data synthesis was exported in format.csv based on the functions available in the “broom” package. For the analysis of the specific resistance potential of each cementitious composite combination to colonization with both species of fungi, the main Component Analysis (PCA) based on the functions of the “vegan” package was applied [79,80,81,82,83].

## 3. Results and Discussions

### 3.1. Assessment of the Ability to Inhibit Mold Development

**Figure 2:**
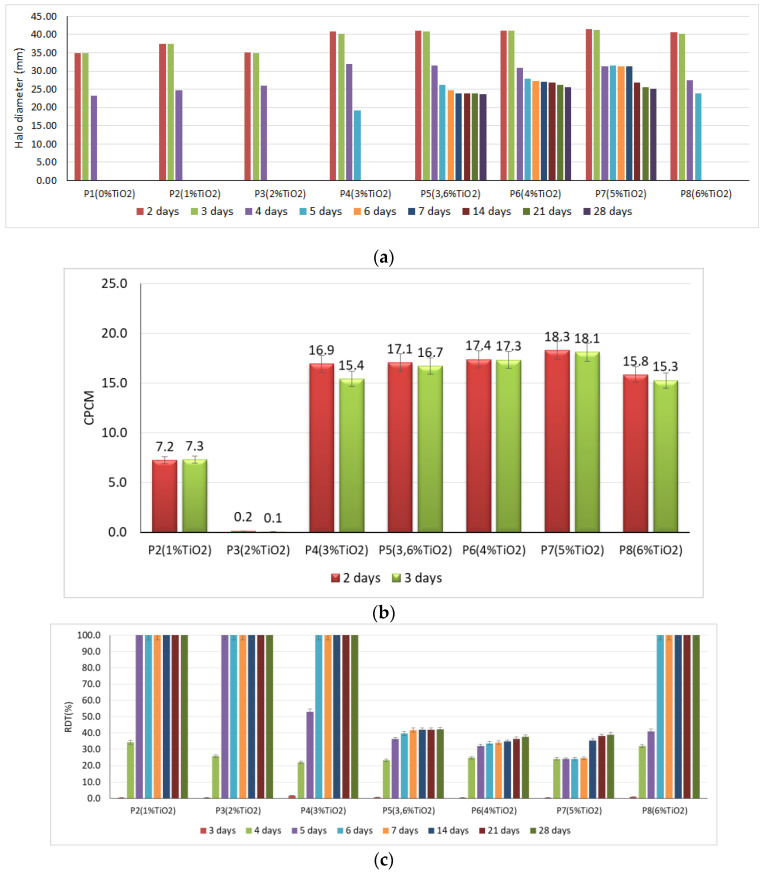
Evolution of the inhibition halo diameter in *Aspergillus niger* exposure: (**a**) time variation of the inhibition halo; (**b**) percentage value, relative to the control sample, which represents the larger the diameter of the inhibition halo compared to the control’s inhibition diameter; (**c**) reduction in the diameter of the inhibition halo over time.

**Figure 3:**
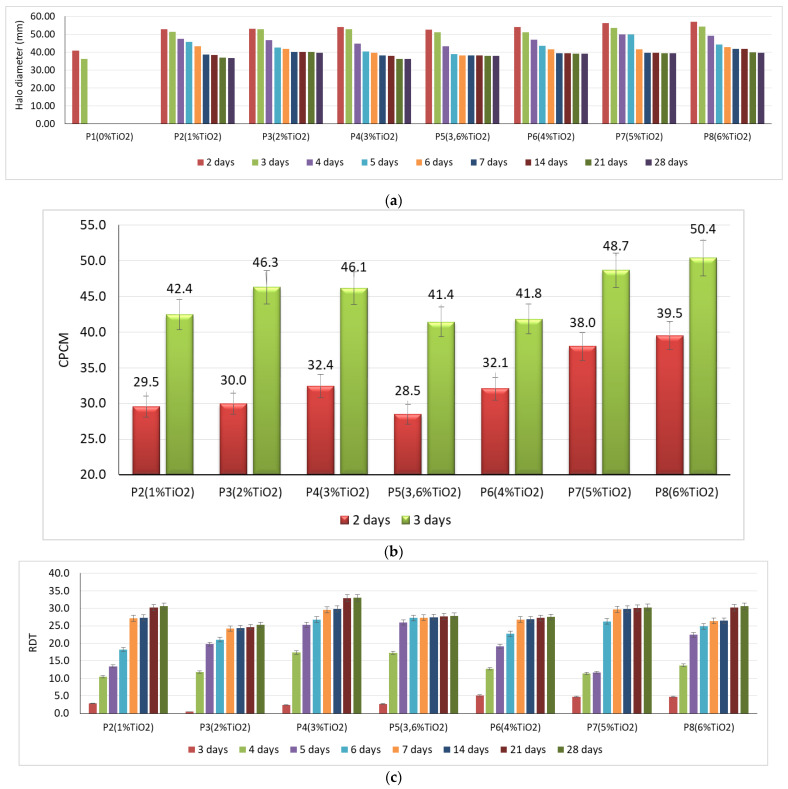
Evolution of the inhibition halo diameter in *Penicillium notatum* exposure: (**a**) time variation of the inhibition halo; (**b**) percentage value, relative to the control sample, which represents the larger the diameter of the inhibition halo compared to the control’s inhibition diameter; (**c**) reduction in the diameter of the inhibition halo over time.

**Figure 4:**
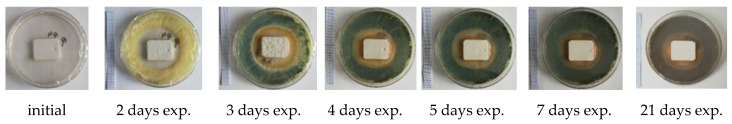
Images of the appearance of the test system (exemplary for the system containing the cementitious composite sample with 3% TiO_2_) in case of exposure to *Penicillium notatum*.

**Figure 5:**
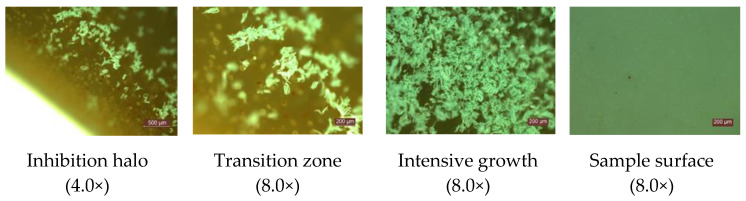
Microscopic analysis (exemplary for system containing cementitious composite sample with 3% TiO_2_) in case of 7 days exposure in environment contaminated with *Penicillium notatum*.

**Figure 6:**
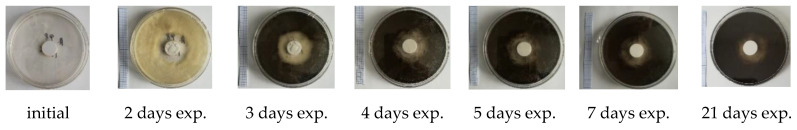
Images of the appearance of the test system (exemplary for the system containing the cementitious composite sample with 5% TiO_2_) in case of exposure to *Aspergillus niger*.

**Figure 7:**
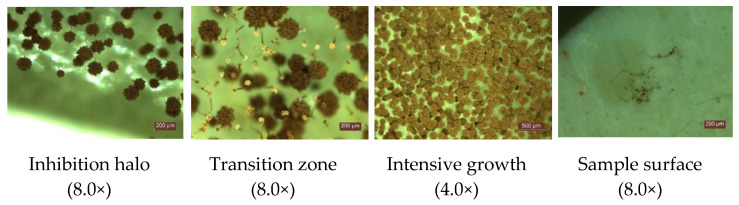
Microscopic analysis (exemplary for system containing cementitious composite sample with 4% TiO_2_) in case of 7 days exposure in environment contaminated with *Aspergillus niger*.

Experimental results on the behavior of the cementitious composites with the addition of TiO_2_ in an environment contaminated with *P. notatum* and *A. niger* spores, respectively, showed that:

- In case of exposure to an environment contaminated with *A. niger* spores in the control sample (0% TiO_2_) and in samples with 1% TiO_2_ and 2% TiO_2_, respectively, the diameter of the inhibition halo (D) decreases, completely disappearing after 5 days of exposure. The extinction of the inhibition halo was also observed on P8 sample (6% TiO_2_) after 6 days of exposure in the contaminated environment. In the case of samples containing 3.6%; 4%; 5% TiO_2_ in the cementitious matrix, the inhibition halo is maintained throughout the evaluation (28 days) in the case of contamination with *A. niger* spores (Figure 2a).
- In the control sample (0% TiO_2_), the diameter of the inhibition halo (D) decreases, completely disappearing after 3 days of exposure, in case of exposure to environment contaminated with *P. notatum* spores (Figure 3a).
- The diameter of the inhibition halo decreases over time, this decrease being stronger in the first 3–4 days of exposure in the contaminated environment, after which a tendency of relative stabilization is observed for both types of mold (Figure 2a and Figure 3a).
- Compared to the control sample (0% TiO_2_), the diameters of the inhibition halo (D) recorded for all nano-TiO_2_-containing cementitious samples are significantly higher, regardless of the duration of exposure in the contaminated environment, indicating good biocidal activity in both mold cases. Relative to the diameter of the control inhibition halo, it is observed that the diameters of the inhibition halo of all samples containing nano-TiO_2_ are greater by 28–38% at 2 days and by 40–50% at 3 days of exposure in the environment contaminated with *P. notatum* spores and by 18% at 2 days and by 15% at 3 days of exposure in the environment contaminated for cementitious samples containing 3–6% nano-TiO_2_, in the case of an environment contaminated with *A. niger* spores (Figure 2b and Figure 3b). After 3 days of exposure, these assessments were no longer performed as the control samples for both *P. notatum* and *A. niger* contamination lost the inhibition halo. However, the subsequent behavior indicates that the biocidal efficiency is not directly proportional to the concentration of nanoparticles and, as a result of the behavior over time, it was assessed that a percentage greater than 5% nano–TiO_2_ in the cementitious matrix is not beneficial, neither economically nor in terms of efficiency. This finding could also be supported by some aspects reported in the literature. On one hand, there are reports showing the difficulty of homogeneous dispersion of nano-TiO_2_ in the cementitious matrix for cases where the amount of nanoparticles introduced is greater, they tend to agglomerate locally and the cementitious composite becomes inhomogeneous in terms of self–cleaning capacity [17,35,59]. On the other hand, by carrying out a parallel with studies on biocidal evolution in general, which indicate that in the case of too high a concentration of biocidal substance, the cell is no longer affected, and it is necessary in most cases to identify the optimal concentration of biocidal substance to obtain a biocidal effect [31,84,85], there is also in the case of these cementitious composites the possibility of too much activity, to which the cells no longer react. By correlating with some references reported in the literature, we can advance the hypothesis that fungi have the ability to bind TiO_2_ particles and fix them in the mycelium. Under conditions of too high concentration of nano-particles, the reaction of the fungi will be of concentration-induced resistance and the performance is no longer achieved [84,85].
- With regard to the evolution over time of the inhibition halo, their diameter decreases for all tested specimens, reducing, compared to the values recorded after exposure 2 days in a contaminated environment, by 2–5% after 3 days, 10–17% after 4 days, up to 27–33% after 28 days exposure in environment contaminated with *P. notatum* spores, highlighting the 2% nano–TiO_2_ composite sample with the smallest decrease halo of inhibition over time (Figure 3c). In case of contamination with *A. niger* spores, the reduction in inhibition halos over time (Figure 2c), evolves according to the duration of exposure and the content of nano–TiO_2_ in the cementitious matrix: samples at which this reduction is total after 5 days (P2, P3) or after 6 days (P4, P8), highlighting the composite samples with 3,6–5% nano–TiO_2_ showing the maintenance of the inhibition halo throughout the evaluation (28 days);
- In the visual analysis, with the naked eye, under the incidence of natural light, on the surface of none of the samples of cementitious material tested there were no traces of mold development, in case of contamination with spores of *P. notatum*, and isolated points with signs of initiation of mold growth after maintenance at least 7 days in an environment contaminated with *A. niger*;
- Microscopic analysis of the surface of the samples, the inhibition halo, the medial areas and the areas removed from the composite sample within the entire test system (Petri dish with PDA growth medium and cementitious composite sample), revealed the presence of three major areas of contamination and growth of the biological material (Figure 4, Figure 5, Figure 6 and Figure 7):
  ◦ The inhibition zone extending circularly around the cementitious composite sample to a maximum distance equal to the diameter of the inhibition halo (D). In this area, the degree of contamination corresponds to the characterization “sterile” or maximum “1–10 colonies of microorganisms”
  ◦ The transition zone in which the biological charge, the density of mold colonies, increases as the distance from the edge of the cementitious composite specimen increases
  ◦ The area of intense growth, in the extremity of the analyzed system, in which the biological load, the density of mold colonies, is so great that independent units can no longer be identified, all being confluent with each other.

### 3.2. Assessment of the Influence of the Shape and Size of the Specimen on the Resistance to Mold Development

**Figure 8:**
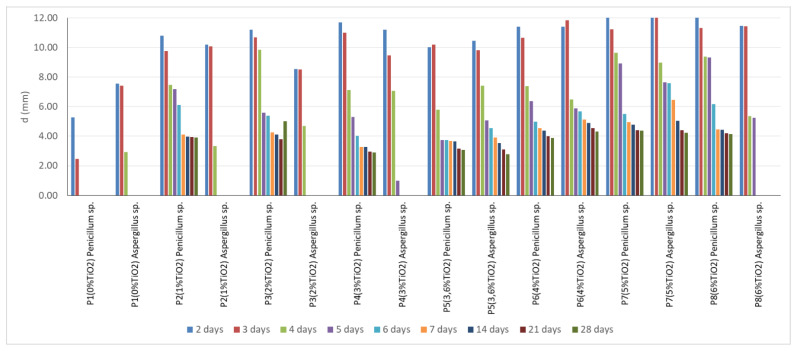
The distance from the edge of the cementitious composite sample to the outer edge of the inhibition halo under conditions of contamination with *Penicillium notatum* and *Aspergillus niger,* respectively.

Measuring the distance from the edge of the cementitious composite sample to the outer limit of the inhibition halo, (d), it was found that, in general, the measurable indicator (d) has a lower value in the case of the environment contaminated with *A. niger* spores, for the same ratio of TiO_2_ introduced in the cementitious matrix (Figure 8). This first observation cannot, however, eloquently indicate that the larger size of the samples subjected to the environment contaminated with *P. notatum* spores would also favor the distance from the edge of the sample on which the inhibition halo extends. It cannot be said for sure that the spores of *A. niger* show a greater aggressiveness, but, nevertheless, this hypothesis can be supported by the identification of specific traces of development of this type of mold on the surface of samples kept for a long time in a contaminated environment, an observation that is not valid for the surface of samples exposed to *P. notatum* spores.

Therefore, it is considered that the shape and dimensions of the tested samples can influence to some extent the quantitative indicators, but certainly cannot influence qualitatively the proof of the mold growth inhibition effect. By correlation with the literature, it is considered desirable to have a disc shape samples, especially for easier measurement of the dimensions characteristic of the inhibition halo, but even samples with other shapes can be tested for a qualitative proof of the anti–mold effect. This hypothesis is supported by the circular shape of the inhibition halo even in the case of rectangular shaped cementitious composite samples.

### 3.3. Data Analysis

Analysis of the capacity to restrict the development of the species *A. niger* (Table 2) highlights the reduction in this feature directly proportional to the length of time. The maximum level of the significant range of variation for the F test is 165.35 (*p* < 0.001) at the beginning of the experiment and is significantly maintained until the 14th day (F = 10.32, *p* value = 0.002). Transposed into average values of the halo, the range of variation is 7 mm between variants, with a maximum for P4–P8 mixtures, compared to P1–P3 mixtures. The differences between these two groups were significant. The same trend is maintained on the third day of experimentation, with an insignificant reduction in the diameter recorded. Starting from the fourth day of experimentation, significant differences can be observed between the tested samples. A halo of more than 30 mm can be observed only for P4–P7 mixtures, the others having values below 28 mm. The differences recorded are significant only when comparing the two groups with each other. An interesting case is mixture P8, which shows a reduction to 27.56 mm in diameter of the halo on this day. From the 5th day of experimentation, samples P1–P3 are completely affected by mold, similarly P8. 

This observation contributes to the support of the hypothesis expressed above indicating the need to identify an optimal concentration range of nano–TiO_2_ in the cementitious matrix to ensure biocidal efficiency, i.e., the quantity of nanoparticles impressing the self–cleaning effect shall be sufficiently large to ensure an obvious and lasting biocidal effect, but not more than the concentration limits to ensure the possibility of homogeneous dispersion in the composite matrix and not induce a possible resistance reaction of the cell. The halo reduction dynamics at this point of the experiment only indicate mixture P7 capable of maintaining the halo above 31 mm, followed by P5 and P6 mixtures, with halos in the range 26–28 mm. Mixture P8 is placed between P5–P7 and P1–P4 mixtures as the level of maintaining the value of the halo.

From the 6th day, only three mixtures maintain their ability to repel *A. niger*. On this day, the P6 sample makes the transition between the P5 and P6 compositions, with significant differences only between these two mixtures. On the 7th day of experimentation, the P7 sample maintains its ability to stop the growth of fungi, but decreases over the next 7 days (day 14) by 5 mm. In the case of P5 and P6 mixtures, the observed restrictions on the diameter of the halo are much smaller and are maintained at a level below 1 mm in an interval of 7 days. From the 21st day of experimentation, no significant differences are observed between the mixtures, all of which maintain the halo at a level above 23–25 mm.

In contrast to the growth dynamics of *A. niger*, in the case of *P. notatum* there is a lower aggressiveness (Table 3). The values of the F test are lower, with significant differences in the range 4.66–41.40. These limits present very interesting cases. In the case of the F test value of 41.40 (day 3) the highest differences between the tested compositions were observed. At the level of 7 days of testing, the F test is reduced to the value of 1.01, all variants tested are similar from the perspective of the observed halo. At 48 h after inoculation, only in the case of P1, a reduction in the halo is observed to almost 40 mm, the differences being significant only in the case of comparison between a mixture of P8 and the other. 

After 24 h (day 3) the halo reduction trend is 1–2 mm, maintaining the differences between the experimental variants. From the 4th day of experimentation, mixture P1 is completely affected by fungi. The differences between the E variants keep small, the meanings being visible only when comparing mixtures P7–P8 with P5. On the 5th day, P7 maintains its repellency capacity, with significant differences from most mixtures except P2. The same mixture shows a sharp 8 mm reduction in the halo in the 24–hour interval between days 5–6, reaching the level of the other mixtures. 

The differences between the experimental variants are significant only when comparing P5 with P8. On the 7th day of experimentation, only mixture P2 undergoes an average reduction in the halo by 5mm. In the case of the other mixtures the reduction is 1–2 mm, but without significant differences. The reductions remain insignificant, generally below the value of 0.5–1 mm/7 days, which indicates the performance of cementitious mixtures in the long term, regardless of the percentage of TiO_2_.

**Figure 9:**
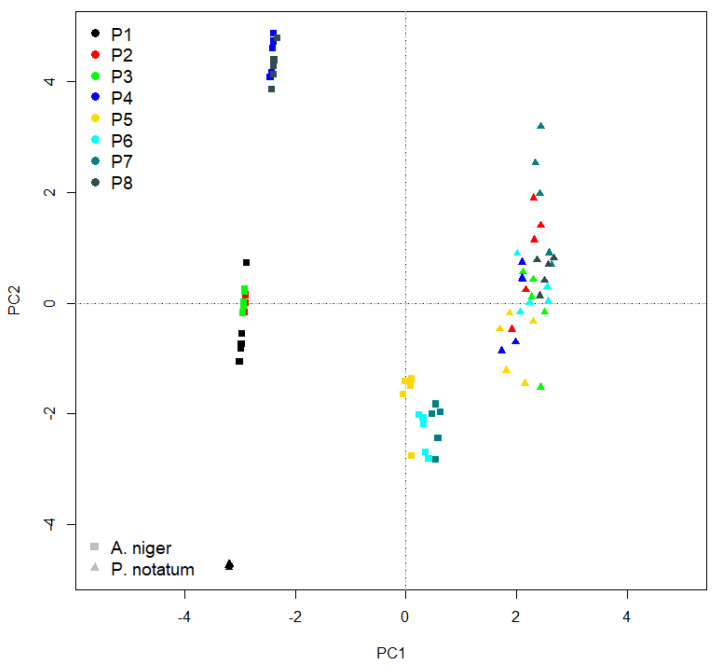
Principal Component Analysis of capacity to restrict the development of fungal colonies due to the concentration of TiO_2_ in cementitious composite. The species used in experiment are *Aspergillus niger (A. niger)* and *Penicillium notatum (P. notatum)*; each of the concentrations are presented as P1–P8, described in Table 1.

The principal components’ analysis (Figure 9) highlights very well the different reaction induced by the percentage of TiO_2_ particles integrated in the cementitious composite. The variance explained by both axes totals 98.57%, with a greater share of Axis 1 (91.39%) compared to Axis 2 (7.19%). In the case of *P. notatum*, the lack of TiO_2_ does not block colonization, the location on the PCA ordering being grouped in the basal part and at approximately equal distance from both axes. This aspect supports the lack of toxicity of cement to the installation of fungi. In the case of the species *A. niger*, the mixtures P5–P7, with high capacity to maintain the non-colonized surface, are highlighted. These three concentrations have the ability to block colony expansion and maintain this mechanism over a period of 4 weeks. Superimposed over Axis 2, in the case of the same species, concentrations of P1–P3 are observed, with reduced ability to block the mechanism of fungal expansion and loss of this property in the first 48–72 h after inoculation.

In the upper part of the ordering are the concentrations of P4 and P8, both of which have an average capacity to block the development of *A. niger*, but which lose this property after a week after inoculation. In the case of *P. notatum*, any concentration of TiO_2_ acts to block colony development up to a diameter of 20–25 mm around the colony. The differences between the concentrations are much smaller in this case, being defining as differentiation the rate of reduction in the protection area.

The similarity between the concentrations of P2 and P7 is given by the ability to maintain the reduction in the halo in the same percentage range, from day to day. The concentrations P3–P4, P6 and P8, respectively, are located in the same area of the ordering; in each of them a value similar to P5 is observed.

## 4. Conclusions

The aim of this work was to analyze the mold inhibition capacity presented by cementitious composites based on white Portland cement with the addition of nano–TiO_2_ and to identify the concentration range of nanoparticles, in relation to the amount of cement, which provides an effective effect from the point of view of biological resistance. For this purpose, two species of mold, *Aspergillus niger* and *Penicillium notatum*, were used for colonization. Based on the presented results, the following can be said:

- All test systems showed three major zones of contamination and growth of biological material: the zone of inhibition halo (in the immediate vicinity of the cementitious sample, with a biological load of maximum 1–10 colonies of microorganisms); the transition zone in which the biological load, the density of mold colonies, increases as the distance from the edge of the cementitious composite specimen increases and the zone of intense growth (at the extremity of the test system, where the biological load is so large that independent colony units can no longer be identified, all of which are confluent with each other).
- No traces of *P. notatum* mold development were observed on the surface of any of the tested cementitious material samples. The surface of the cementitious composites exposed in an environment contaminated with *A. niger* showed isolated spots with signs of mold growth initiation, after conditioning for at least 7 days of testing.
- As the duration of exposure of the cementitious samples in the contaminated environment increases, depending on the content of nano–TiO_2_, the dimensions of the inhibition halo change to reduce them. Thus, in the case of *A. niger* it disappears completely after 5 days of testing for the control sample (P1) and for the mixtures P2 and P3 (with 1% TiO_2_ and 2% TiO_2_, respectively) and after 6 days of testing for the sample P8 (6% TiO_2_). For P3, P4 and P5 composites (3.6%; 4%; 5% TiO_2_) the inhibition halo is maintained throughout the testing period (28 days), showing dimensional reductions in the first 3–4 days, after which a relative stabilization is observed. In case of exposure to *P. notatum* spores contaminated environment the control sample P1 (0% TiO_2_) loses the inhibition halo after 3 days of testing but all TiO_2_-containing cementitious composites (P2–P8) the inhibition halo is maintained throughout the test.
- The mix–design of cementitious composites mixtures included two extreme variants of nano–TiO_2_ content, respectively P1 (0% nano–TiO_2_) and P8 (6% nano–TiO_2_) following several hypotheses:
  ◦ Thus, (I) the hypothesis of the biocidal effect of photoactivation of nanoparticles in the cementitious matrix was confirmed based on the comparison of the characteristics of inhibition haloes compared to the control sample, P1 (0% nano-TiO_2_).
  ◦ The hypothesis of increasing the proportional and continuous biocidal effect, with the increase in the content of nano–TiO_2_ in the cementitious matrix (II) was disproved by identifying the maximum functionality threshold, respectively, P7 (5% nano–TiO_2_), and by the less efficient behavior of the composite P8 (6% nano–TiO_2_). The behavior of this excess nanoparticle composite can be accepted on the basis of two hypotheses extracted in accordance with the specifications of the literature and which will be developed in future studies: (1) the difficulty of homogeneous dispersion of a large amount of nanoparticles in the cementitious matrix which results in the inhomogeneity of the composite with the existence of agglomeration areas of nanoparticles and areas with insufficient nano–TiO_2_ content and (2) the possibility that, some known biocidal treatment solutions, too high a concentration causes the cell to become insensitive. Analyzing CPCM and RTD in the case of *P. notatum*, a slight increase in this indicator for the analyzed mixtures was observed when using 6% nano-TiO_2_ addition, compared to 5%. However, it was assessed that this increase in the efficiency of the biocide effect is not necessarily sufficiently motivating for an increase in the concentration of nano–TiO_2_ to 6%, which also attracts an increase in production costs. Moreover, it can be said that for different species of mold, the effectiveness of the addition of TiO_2_ nanoparticles reacts differently and detailed research is needed for each individual case.
  ◦ In addition, the hypothesis that the introduction of some amount of nano–TiO_2_ into the cementitious matrix automatically induces a capacity for resistance to mold growth, regardless of its species Type (III) is disproved by two observations as follows: (1) an insufficient amount of nanoparticles does not ensure the maintenance of the inhibition halo (see behavior of mixtures P2 (1% TiO_2_), P3 (2% TiO_2_) exposed in a contaminated environment with *A. niger*) and therefore does not ensure the durability of the biocidal effect and (2) the resistance to mold development differs according to its species, as demonstrated by comparing the behavior of composites with the same content of nano-TiO_2_ exposed in each of the two contaminating media.
- The differentiation of the shape/dimensions of the tested samples contributed to the evaluation of the acceptability of the hypothesis (IV) that considers that the geometric aspects of the tested cementitious composite sample are defining for the quantitative and qualitative indicators of evaluation of the biocidal effect. In this sense, it can be said that the geometric aspects influence to some extent the quantitative indicators, but, certainly, cannot influence qualitatively the proof of the effect of inhibition of mold growth, a statement supported by the circular shape of the inhibition halo (the most obvious qualitative initiator) even in the case of cementitious composite samples with rectangular shape. However, in order to facilitate and increase the accuracy of measurements and quantitative evaluation, a disc shape of the test specimens was considered desirable.
- Statistical analysis of quantitative performance evaluation indicators (inhibition halo dimensions) was a useful tool for supporting decisions to confirm or refute the previously mentioned hypotheses. The results obtained experimentally and analyzed statistically open a new direction in the continuation of experimental researches aimed at maintaining the ability to inhibit the development of fungi even after the action of the aging process, the small concentrations having the chance of not inducing resistance to biocide action over time.

As a result of those presented, it can be appreciated that in the case of cementitious composites with nano-TiO_2_ content, in addition to the self-cleaning effect demonstrated by numerous researches reported in the literature, the increased and lasting resistance to mold development is a proven property, which, however, is dependent on both the compositional characteristics of the material and the mold species. As such, it is considered necessary to continue the development of the experimental research in this direction, which are both types of cement, and the nanoparticles are different from those used in the present study, as well as other species of fungi, algae, fungi, etc., the development of which it is commonly found on the surface of the built environment, all of which contribute to the increase in the safety and health of the population, as well as to increase the sustainability of the construction industry and, consequently, to the reduction in the pollution of the environment.

## Figures and Tables

**Table 1:** Mixdesign ratio and conditioning of the samples.

Mixture	P1	P2	P3	P4	P5	P6	P7	P8
Amount of Nanoparticles Relative to The Amount of Cement (%)	0	1	2	3	3.6	4	5	6
CEM I 52.5R White Cement, HOLCIM ROMANIA (g)	500	500	500	500	500	500	500	500
Quantity of Water Relative to Quantity of Total Dry Mixture (water/(cement + nano–TiO_2_)) (g)	0.45–0.50							
Conditioning	24 h in Molds, 90% RH, 20 °C, without Light; Demolding. 27 Days of Total Immersion in Water, 20 °C, without Light; Until Photoactivation Followed by Testing, under Laboratory Conditions, without Light							

**Table 2:** Analysis of the capacity to restrict the development of the species *Aspergillus niger*.

	2 Days Exposure	3 Days Exposure	4 Days Exposure	5 Days Exposure	6 Days Exposure	7 Days Exposure	14 Days Exposure	21 Days Exposure	28 Days Exposure
P1	34.98 ± 0.23 ^c^	34.89 ± 0.32 ^c^	23.25 ± 1.52 ^c^						
P2	37.51 ± 0.22 ^b^	37.44 ± 0.44 ^bc^	24.64 ± 0.39 ^bc^						
P3	35.04 ± 0.13 ^c^	34.92 ± 0.64 ^c^	25.99 ± 0.34 ^bc^						
P4	40.9 ± 0.37 ^a^	40.27 ± 1.06 ^ab^	31.86 ± 0.65 ^b^	19.25 ± 0.02 ^d^					
P5	40.95 ± 0.13 ^a^	40.73 ± 0.78 ^a^	31.44 ± 0.62 ^a^	26.11 ± 0.74 ^bc^	24.69 ± 1.05 ^b^	23.81 ± 1.25 ^b^	23.79 ± 0.48 ^b^	23.79±1.03 ^a^	23.69 ± 1.04 ^a^
P6	41.06 ± 0.26 ^a^	40.94 ± 0.46 ^a^	30.89 ± 0.32 ^a^	27.88 ± 0.59 ^b^	27.27 ± 1.56 ^ab^	27.02 ± 1.12 ^b^	26.78 ± 0.32 ^a^	26.12±0.29 ^a^	25.64 ± 0.38 ^a^
P7	41.37 ± 0.12 ^a^	41.21 ± 0.81 ^a^	31.37 ± 0.25 ^a^	31.35 ± 0.78 ^a^	31.38 ± 1.02 ^a^	31.23 ± 0.58 ^a^	26.73 ± 0.72 ^a^	25.53±0.84 ^a^	25.22 ± 0.73 ^a^
P8	40.52 ± 0.13 ^a^	40.22 ± 0.6 ^ab^	27.56 ± 0.46 ^a^	23.87 ± 0.76 ^c^					
F. test	165.35	15.78	25.41	49.35	7.45	13.20	10.32	2.37	1.78
*p* val	0.000	0.000	0.000	0.000	0.008	0.001	0.002	0.136	0.210

* Note: Means ± s.e. followed by different letters indicates significant differences between variants at *p* < 0.05 according to LSD Test.

**Table 3:** Analysis of the capacity to restrict the development of the species *Penicillium notatum*.

	2 Days Exposure	3 Days Exposure	4 Days Exposure	5 Days Exposure	6 Days Exposure	7 Days Exposure	14 Days Exposure	21 Days Exposure	28 Days Exposure
P1	40.83 ± 1.46 ^c^	36.1 ± 1.22 ^b^							
P2	52.89 ± 0.65 ^ab^	51.42 ± 0.87 ^a^	47.37 ± 1.41 ^ab^	45.79 ± 1.75 ^ab^	43.27 ± 1.42 ^a^	38.53 ± 1.93 ^a^	38.46 ± 0.3 ^b^	36.91 ± 0.79 ^a^	36.69 ± 0.83 ^a^
P3	53.06 ± 1.44 ^ab^	52.8 ± 0.75 ^a^	46.82 ± 1.29 ^ab^	42.6 ± 0.63 ^bcd^	41.89 ± 0.62 ^ab^	40.23 ± 1.27 ^a^	40.16 ± 0.27 ^ab^	40.04 ± 1.02 ^a^	39.68 ± 0.97 ^a^
P4	54.07 ± 0.57 ^ab^	52.76 ± 0.93 ^a^	44.7 ± 1.52 ^ab^	40.42 ± 1.16 ^cd^	39.58 ± 1 ^ab^	38.09 ± 0.9 ^a^	37.96 ± 0.88 ^b^	36.31 ± 1.18 ^a^	36.25 ± 1.05 ^a^
P5	52.46 ± 0.78 ^b^	51.05 ± 0.7 ^a^	43.41 ± 0.96 ^b^	38.86 ± 0.98 ^d^	38.18 ± 0.66 ^b^	38.14 ± 1.5 ^a^	38.07 ± 0.68 ^b^	37.94 ± 1.24 ^a^	37.86 ± 1.26 ^a^
P6	53.93 ± 0.32 ^ab^	51.19 ± 1.25 ^a^	47.07 ± 1.01 ^ab^	43.61 ± 1.31 ^bcd^	41.67 ± 0.89 ^ab^	39.49 ± 0.92 ^a^	39.45 ± 0.34 ^ab^	39.24 ± 1.61 ^a^	39.1 ± 1.64 ^a^
P7	56.33 ± 0.8 ^ab^	53.67 ± 0.64 ^a^	49.91 ± 0.78 ^a^	49.8 ± 0.65 ^a^	41.54 ± 1.37 ^ab^	39.62 ± 1.87 ^a^	39.57 ± 1.12 ^ab^	39.4 ± 1.1 ^a^	39.29 ± 1.07 ^a^
P8	56.96 ± 0.22 ^a^	54.28 ± 0.78 ^a^	49.18 ± 0.54 ^a^	44.2 ± 0.63 ^bc^	42.83 ± 0.41 ^a^	41.94 ± 0.54 ^a^	41.9 ± 0.37 ^a^	39.78 ± 0.89 ^a^	39.55 ± 0.74 ^a^
F.test	31.57	41.40	4.17	10.83	3.41	1.01	4.66	1.65	1.62
p.val	0.000	0.000	0.004	0.000	0.012	0.441	0.002	0.170	0.180

* Note: Means ± s.e. followed by different letters indicates significant differences between variants at *p* < 0.05 according to LSD Test.

## Data Availability

The data presented in this study are available on request from the corresponding authors.
